# Transmitted drug resistance and transmission clusters among ART-naïve HIV-1-infected individuals from 2019 to 2021 in Nanjing, China

**DOI:** 10.3389/fpubh.2023.1179568

**Published:** 2023-08-22

**Authors:** Yuanyuan Xu, Hongjie Shi, Xiaoxiao Dong, Chengyuan Ding, Sushu Wu, Xin Li, Hongying Zhang, Mengkai Qiao, Xiaoshan Li, Zhengping Zhu

**Affiliations:** ^1^Department of AIDS/STD Control and Prevention, Nanjing Center for Disease Control and Prevention, Nanjing, China; ^2^Department of Microbiology Laboratory, Nanjing Center for Disease Control and Prevention, Nanjing, China; ^3^Center for Global Health, School of Public Health, Nanjing Medical University, Nanjing, China; ^4^Department of Lung Transplant Center, The Affiliated Wuxi People’s Hospital of Nanjing Medical University, Wuxi People’s Hospital, Wuxi Medical Center, Nanjing Medical University, Wuxi, China

**Keywords:** HIV, transmitted drug resistance, mutations, transmission cluster, antiretroviral therapy

## Abstract

**Background:**

Transmitted drug resistance (TDR) is an increasingly prevalent problem worldwide, which will significantly compromise the effectiveness of HIV treatments. However, in Nanjing, China, there is still a dearth of research on the prevalence and transmission of TDR among ART-naïve HIV-1-infected individuals. This study aimed to understand the prevalence and transmission of TDR in Nanjing.

**Methods:**

A total of 1,393 participants who were newly diagnosed with HIV-1 and had not received ART between January 2019 and December 2021 were enrolled in this study. HIV-1 *pol* gene sequence was obtained by viral RNA extraction and nested PCR amplification. Genotypes, TDR and transmission cluster analyses were conducted using phylogenetic tree, Stanford HIV database algorithm and HIV-TRACE, respectively. Univariate and multivariate logistic regression analyses were performed to identify the factors associated with TDR.

**Results:**

A total of 1,161 sequences were successfully sequenced, of which CRF07_BC (40.6%), CRF01_AE (38.4%) and CRF105_0107 (6.3%) were the main HIV-1 genotypes. The overall prevalence of TDR was 7.8%, with 2.0% to PIs, 1.0% to NRTIs, and 4.8% to NNRTIs. No sequence showed double-class resistance. Multivariate logistic regression analysis revealed that compared with CRF01_AE, subtype B (OR = 2.869, 95%CI: 1.093–7.420) and female (OR = 2.359, 95%CI: 1.182–4.707) were risk factors for TDR. Q58E was the most prevalent detected protease inhibitor (PI) -associated mutation, and V179E was the most frequently detected non-nucleoside reverse transcriptase inhibitor (NNRTI) -associated mutation. A total of 613 (52.8%) sequences were segregated into 137 clusters, ranging from 2 to 74 sequences. Among 44 individuals with TDR (48.4%) within 21 clusters, K103N/KN was the most frequent TDR-associated mutation (31.8%), followed by Q58E/QE (20.5%) and G190A (15.9%). Individuals with the same TDR-associated mutations were usually cross-linked in transmission clusters. Moreover, we identified 9 clusters in which there was a transmission relationship between drug-resistant individuals, and 4 clusters in which drug-resistant cases increased during the study period.

**Conclusion:**

The overall prevalence of TDR in Nanjing was at a moderate level during the past 3 years. However, nearly half of TDR individuals were included in the transmission clusters, and some drug-resistant individuals have transmitted in the clusters. Therefore, HIV drug-resistance prevention, monitoring and response efforts should be sustained and expanded to reduce the prevalence and transmission of TDR in Nanjing.

## Introduction

HIV/AIDS remains a major public health problem in China. The number of people living with HIV/AIDS (PLWH) in Nanjing has been continuously increasing in recent years. As of 2022, there were 6, 564 PLWH. Nanjing, as the capital of Jiangsu Province, is located in east of China and has the second largest number of PLWH in the province. The widespread coverage of antiretroviral therapy (ART) has reduced HIV-associated morbidity and mortality, and has substantially curbed rampant HIV transmission (1, 2). However, with efforts underway to expand ART and pre-exposure prophylaxis (PrEP), as well as more individuals receive antiretroviral drugs for treating and preventing HIV, HIV drug resistance may further increase (3, 4). HIV drug resistance could significantly compromise the effectiveness of HIV treatments, leading to possible increase in HIV incidence and HIV-associated morbidity and mortality (5).

According to the definition of the World Health Organization (WHO), HIV drug resistance is classified into three main categories: acquired HIV drug resistance (ADR), transmitted drug resistance (TDR) and pretreatment HIV drug resistance (PDR) (6). Transmitted HIV drug resistance will occur when individuals are infected with HIV with drug-resistance mutations. Several studies have revealed that TDR of HIV is prevalent but varies in different areas worldwide. For example, the TDR prevalence in German is reported to be 17.2% (7), 4.1% in South/Southeast Asia, 6.0% in sub-Saharan Africa and 9.1% in Latin America/Caribbean (8). A nationwide cross-sectional survey conducted in 2015 revealed the overall prevalence of TDR was 3.6% in China (9). Moreover, some cities reported relatively high TDR prevalence in recent years (10), such as 12.2% in Tianjin (11) and 17.4% in Shanghai (12). TDR may lead to the failure of ART and the spread of drug-resistant strains, posing a challenge to the battle against HIV. Therefore, TDR should be an intensive focus of HIV surveillance to prevent the spread of drug-resistant strains.

Molecular transmission network technology is used to identify the maximum number of clusters and links based on the genetic distance threshold using *pol* gene sequences (13, 14). When combined with an epidemiological investigation, the genetic relatedness of HIV-1 could partially reflect the unobserved relationship and the spread of TDR among PLWH (15). Analysis of the molecular transmission networks among newly diagnosed PLWH is conducive to understanding TDR and providing insight for ART and HIV prevention strategies (16).

In Nanjing, HIV prevalence among the general population is below 0.07%, but high in key risky population. For example, we reported over 10% HIV prevalence among men who have sex with men (MSM) (17). Among HIV positive cases newly reported in 2022, homosexual transmission, heterosexual transmission and injecting drug transmission accounted for 64.8, 34.7, and 0.5%, respectively. The rate of ADR among patients experiencing failure of ART exceeded 50% in Jiangsu province, and the rate still rising (18). In addition, The PDR rate is 16.3% among 1, 210 patients who initiated ART and the TDR rate was closed to 5% in 2017 (19, 20). The prevalence and transmission of TDR vary across different geographical regions. Given the alarming drug resistance status in Jiangsu Province, urgent attention needs to be paid to master the status of TDR in Nanjing city. However, there is little information available regarding the prevalence of TDR in Nanjing, as well as the specific patterns of its transmission in molecular networks. Thus, we conducted a consecutive cross-sectional study among ART-naïve HIV-1-infected individuals in Nanjing from 2019 to 2021 and aimed to understand the prevalence of TDR and further elucidate its transmission patterns.

## Materials and methods

### Study subjects and data collection

In our study, 1, 393 ART-naïve individuals newly diagnosed with HIV-1 were enrolled between January 1, 2019 and November 31, 2021. Each patient met the following criteria: (1) Age ≥ 16 years; (2) Confirmed diagnosis of HIV-1 infection; (3) No history of ART. Participants were enrolled at the voluntary counseling testing clinics (VCT) in Nanjing. This study was approved by the Ethics Committee of Nanjing Center for Disease Control and Prevention (Approval ID: PJ2020-A001-03). After the written informed consent obtained from each eligible participants, plasma samples were collected and basic epidemiological such as age, gender, marital status and self-reported transmission routes were recorded upon enrollment.

### RNA extraction, nested-PCR, and sequencing of viral DNA

Within 12 h after blood collection, plasma was isolated and kept at −80°C until analysis. Viral RNA was extracted from 200 μl plasma samples using the QIAamp Viral RNA Mini Kit (Qiagen, Hilden, Germany). The target fragment of 1,059 bp in the pol gene (HXB2:2253–3,312) was amplified using nested polymerase chain reaction (PCR). PrimeScriptTM One Step RT-PCR Ver. 2.0 (TakaRa, China) was used for the first-round PCR procedure and cDNA synthesis. Nested PCR was performed with Ex Taq (TaKaRa, China). The PCR products were sent to Sangon Biotechnology Co., Ltd. for automatic DNA sequencing using ABI 3730XL. The PCR protocol and primers used were as previously described (21).

### Subtype analysis

Sequencer 4.10.1 (GeneCodes, Ann Arbor, MI) was used for sequence splicing, and the secondary peak threshold was set to 30% to identify ambiguities. The sequence was aligned with the reference sequences using BioEdit (version 7.0.9, Informer Technologies Inc.). Reference sequences were downloaded from the Los Alamos HIV database and included the major international epidemic strains A-D, F-H, and J-K, as well as the major epidemic recombinant strains in China. A phylogenetic tree with the maximum likelihood (ML) method was constructed for confirmation using the FastTree 2.1. The nucleotide substitution model was GTR + G + I, and support values were calculated by Shimodaira Hasegawa-like test. Clusters with a bootstrap value higher than 0.90 (90%) were defined as the same subtype. The ML phylogenetic tree was imported to FigTree v 1.4.4 for visualization.

### Drug resistance analysis

We assessed clinically relevant resistance to protease inhibitors (PIs), nucleoside reverse transcriptase inhibitors (NRTIs), or non-nucleoside reverse transcriptase inhibitors (NNRTIs) using the Stanford University HIV Drug Resistance Database Genotypic Resistance Interpretation Algorithm (version 8.8) and the International Antiviral Society Drug Resistance Mutation list (12). The degree of drug resistance to each ART drug was divided into five levels: susceptible (S, 0–9), potential low resistance (PLR, 10–14), low-level resistance (LR, 15–29), intermediate-level resistance (IR, 30–59), and high-level resistance categories (HR, ≥60). TDR was detected among ART-naïve individuals, and sequences classified as low-, intermediate-, or high-level resistance were designated as drug resistance according to the WHO drug resistance report (6).

### Transmission clusters construction

Sequences <1,000 bp in length or containing ≥5% ambiguities were excluded. HIV-TRACE (localized HIV-TRACE, built CentOS 7 platform, wrote program offline) was used to determine the optimal genetic distance threshold and construct molecular network (22). With the optimal threshold of 1.5% genetic distance, the HIV *pol* gene sequence was compared with the reference sequence, and the genetic distances of paired genes were calculated using the Tamura-NEI 93 model to construct the molecular network of TDR transmission (23). Cytoscape (version 3.6.1) was used to process and generate the molecular network.

### Statistical analysis

SPSS (version 18.0, LEAD Technologies Inc.) was used for data analysis. Quantitative data were represented as means ± standard deviation or median (inter-quartile range, IQR). Categorical variable were represented as frequency and percentage, and analyzed using the chi-square test. Linear-by-linear chi-square tests were performed to analyze the trend in TDR prevalence from 2019 to 2021. Univariate and multivariate logistic regression analyses were performed to identify the factors associated with TDR. Variables with a *p* < 0.05 in univariate analyses were included in stepwise multivariate regression analysis. All results of statistical significance test were reported as *p*-values. *p <* 0.05 (two-tailed) was considered statistically significant.

## Results

### Demographic and social characteristics

We sequenced and analyzed 1,161 samples obtained from 1,393 HIV-1 infected individuals in our research. The median age was 29 years (IQR: 24–43 years), raging from16 to 89 years. The majority of the participants were single (62.4%), while the Han majority accounted for 97.9%. In terms of the education, 59.9% were college and higher, and 22.1% were junior or below. The major transmission route was homosexual transmission (68.9%), followed by heterosexual transmission (30.2%; Table 1).

**Table 1:** Demographic characteristics and factors associated with TDR.

Variables	*N* (%)	PDR (%)	OR (95%CI)	*P-*value	Adjusted OR (95%CI)	*P*-value
Sex						
Male	1,090(93.9)	80(7.3)	1.000		1.000	
Female	71(6.1)	11(15.5)	2.315(1.170–4.578)	0.016	2.359(1.182–4.707)	0.015
Age (yrs)						
16–24	346(29.8)	28(8.1)	1.000			
25–49	595(51.3)	50(8.4)	1.042(0.643–1.689)	0.868		
≥50	220(18.9)	13(5.9)	0.713(0.361–1.409)	0.331		
Marital status						
Single	724(62.4)	56(7.7)	1.000			
Married	336(28.9)	28(8.3)	1.084(0.676–1.741)	0.737		
Divorced/widowed	101(8.7)	7(6.9)	0.888(0.393–2.007)	0.776		
Ethnicity						
Han	1,137(97.9)	88(7.7)				
Ethnic minorities	24(2.1)	3(12.5)	1.703(0.498–5.821)	0.396		
Education						
Junior or below	257(22.1)	26(10.1)	1.000			
Senior	209(18.0)	15(7.2)	0.687(0.354–1.334)	0.267		
College or above	695(59.9)	50(7.2)	0.689(0.419–1.132)	0.142		
Transmission route						
Homosexual	800(68.9)	61(7.6)	1.000			
Heterosexual	350(30.2)	29(8.3)	1.094(0.690–1.736)	0.701		
Other	11(0.9)	1(9.1)	1.211(0.153–9.621)	0.856		
Diagnosed with STDs except for HIV						
No	812(70.0)	59(7.3)	1.000			
Yes	201(17.3)	17(8.5)	1.179(0.671–2.071)	0.566		
Unknown	148(12.7)	15(10.1)	1.439(0.793–2.612)	0.231		
Screening source						
VCT	610(52.5)	49(8.0)	1.000			
Medical institution	325(28.0)	23(7.1)	0.872(0.521–1.459)	0.602		
Sexually transmitted disease clinic	130(11.2)	8(6.2)	0.751(0.347–1.626)	0.467		
Other	96(8.3)	11(11.5)	1.482(0.741–2.962)	0.266		
Baseline CD4 cell count (cells/μL)						
<200	260(22.4)	21(8.1)	1.000			
200–499	654(56.3)	52(8.0)	0.983(0.580–1.668)	0.95		
≥500	247(21.3)	18(7.3)	0.895(0.465–1.722)	0.739		
Baseline viral load before ART (copies/ml)						
<10,000	266(22.9)	26(9.8)	1.000			
10,000–99,999	608(52.4)	49(8.1)	0.809(0.491–1.333)	0.406		
≥100,000	287(24.7)	16(5.6)	0.545(0.285–1.040)	0.066		
Subtype						
CRF01_AE	446(38.4)	30(6.7)	1.000		1.000	
CRF07_BC	471(40.6)	38(8.1)	1.217(0.740–2.001)	0.439	1.174(0.712–1.935)	0.530
CRF105_0107	73(6.3)	7(9.6)	1.471(0.621–3.485)	0.381	1.504(0.633–3.569)	0.355
Subtype B	35(3.0)	6(17.1)	2.869(1.105–7.448)	0.030	2.847(1.093–7.420)	0.032
CRF55_01B	36(3.1)	3(8.3)	2.521(0.816–7.789)	0.108	1.332(0.385–4.602)	0.651
Other	100(8.6)	6(5.5)	0.800(0.324–1.973)	0.628	0.968(0.410–2.286)	0.941
Year						
2019	348(30.0)	28(8.0)	1.000			
2020	312(26.9)	24(7.7)	0.952(0.540–1.681)	0.866		
2021	501(43.1)	39(7.8)	0.965(0.582–1.600)	0.889		

TDR, transmitted drug resistance; OR, odds ratio; CI, confidence interval; STDs, sexually transmitted diseases; ART, antiretroviral therapy; CRF, circulating recombinant form.

### Distribution of HIV-1 subtypes

**Figure 1:**
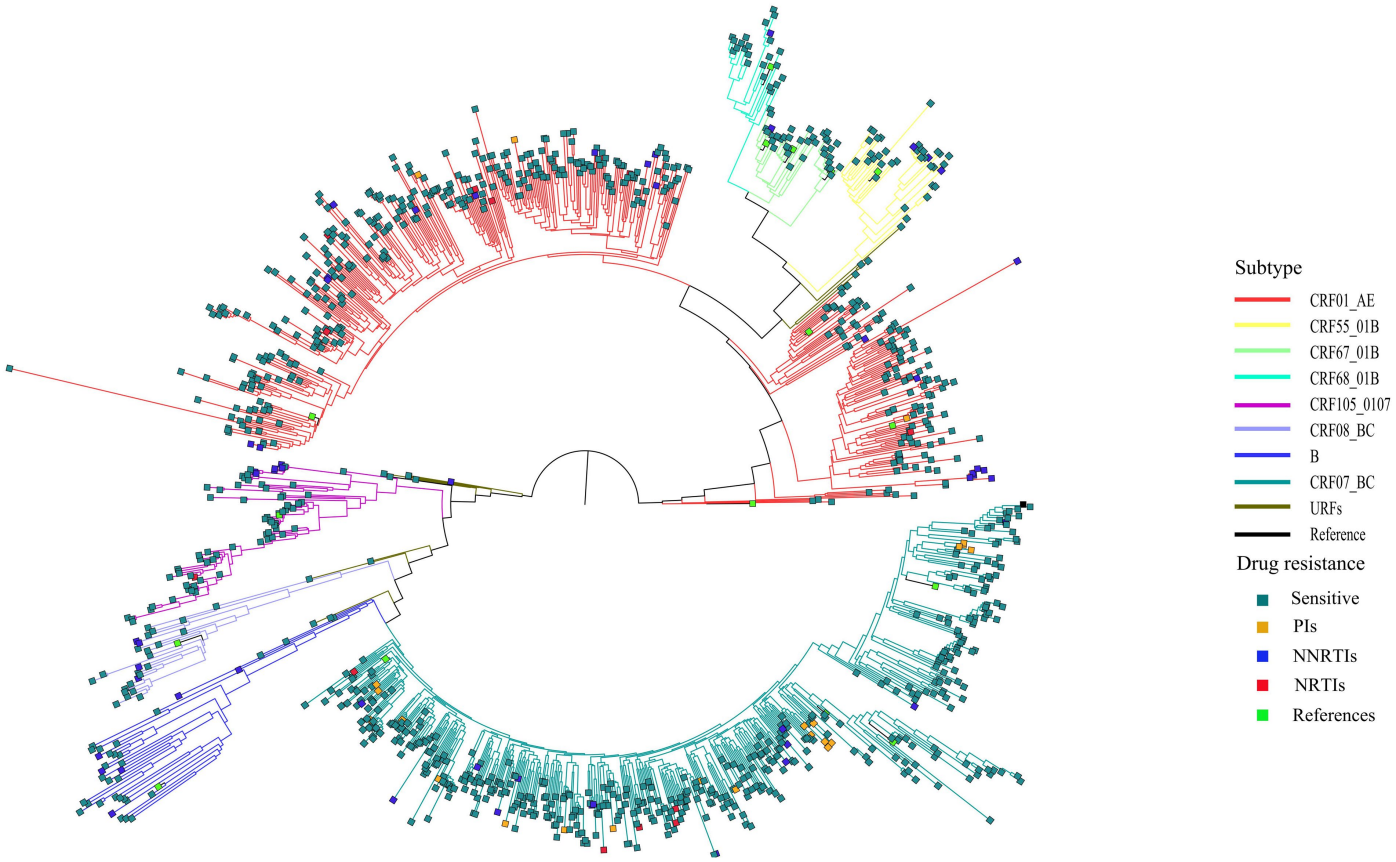
Phylogenetic tree analysis of nucleotide sequences from 1,161 newly diagnosed individual. CRF, circulating recombinant form; URFs, unique recombinant forms; PIs, protease inhibitors; NRTIs, nucleoside reverse transcriptase inhibitors; NNRTIs, non-nucleoside reverse transcriptase inhibitors.

Among the 1,161 successfully amplified sequences, circulating recombinant form (CRF) 07_BC (40.6%) was the predominant subtype, followed by CRF01_AE (38.4%), CRF105_0107 (6.3%), CRF67_01B (3.2%), CRF55_01B (3.1%), subtype B (3.0%), CRF08_BC (2.2%) and CRF68_01B (2.1%). Additionally, another 13 (1.1%) HIV-1 strains did not cluster with any present known reference sequences, and were determined as unique recombinant forms (URFs; Figure 1).

### Prevalence of TDR and the factors associated with TDR

The overall prevalence of transmitted drug resistance was 7.8% (91/1161), including 2.0% (23/1161) to PIs, 1.0% (12/1161) to NRTIs, and 4.8% (56/1161) to NNRTIs. All the HIV-1 strains with TDR displayed a single drug class resistance mutation. From 2019 to 2021, the prevalence of TDR was stable (*χ*^2^ = 0.016, *p =* 0.898), with annual rates of 8.0, 7.8, and 7.8%, respectively. Univariate and multivariate logistic regression analyses showed that compared with CRF01_AE, infection with the Subtype B (aOR = 2.847, 95%CI: 1.093–7.420) and female (aOR = 2.359, 95%CI: 1.182–4.707) were risk factors for TDR (Table 1).

### Analysis of HIV drug resistance mutation sites with subtype

**Figure 2:**
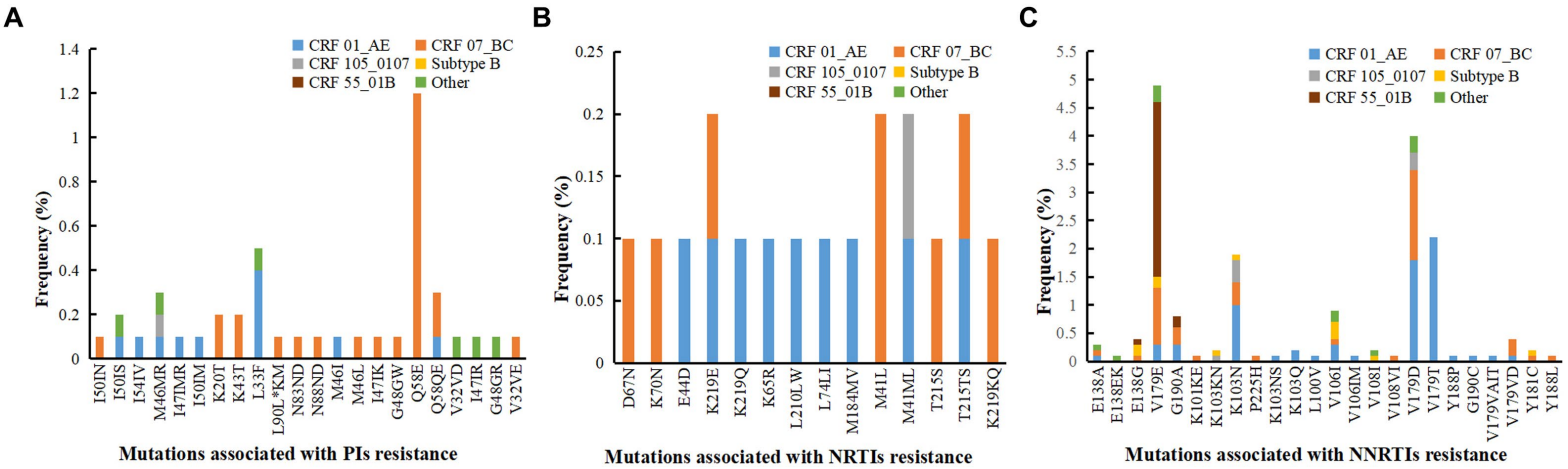
Analysis of drug resistance mutation sites with different subtype. **(A)** Mutations associated with PIs resistance. **(B)** Mutations associated with NRTIs resistance. **(C)** Mutations associated with NNRTIs resistance. PIs, protease inhibitors; NRTIs, nucleoside reverse transcriptase inhibitors; NNRTIs, non-nucleoside reverse transcriptase inhibitors.

Among the 91 drug-resistant individuals, we found 60 kinds of DRM; 24 were associated with resistance to NNRTIs, 14 to NRTIs, and 22 to PIs. Individuals infected with CRF07_BC were most likely to develop PI-related mutations. Q58E (1.2%) were the most prevalent detected PI-associated mutation, and all harbored in CRF07_BC. Individuals infected with CRF01_AE and CRF07_BC were most likely to develop NRTI-associated mutations. The mutation sites primarily comprised M41L (0.2%), M41ML (0.2%), K219E (0.2%) and T215TS (0.2%). Individuals infected with CRF01_AE were most likely to develop NNTRI-associated mutations, followed by CRF07_BC; and V179E (4.9%), V179D (4.0%), V179T (2.2%) and K103N (2.0%) were the most common mutations. Most V179E mutations were detected in CRF55_01B, most V179D mutations were detected in CRF01_AE and CRF07_BC, and all the V179T mutations were detected in CRF01_AE (Figure 2).

### Level of resistance to different antiretroviral drugs

The HIV-1 resistance level to a total of 20 common antiretroviral drugs was analyzed based on the drug resistant mutation score. PIs and NRTIs TDR present mostly low-level resistance. Moreover, NNRTI displayed mainly as intermediate-to high-level resistance, except for ETR and RPV, which exhibited primarily LR (8, 0.7% for ETR; 19, 1.6% for RPV; Table 2). For PIs, TPV showed high TDR frequency (1.6%) and the degree of resistance was all at LR (1.6%) As for NRTIs, D4T (0.9%) and AZT (0.8%) presented high TDR frequency and the degree of resistance was mainly at LR (0.6% for D4T, 0.7% for AZT). As for NNRTIs, NVP (4.3%) and EFV (4.0%) showed high TDR frequency and the degree of resistance was mainly at HR (3.3% for NVP, 2.6% for EFV). A total of 38 cases were high-level resistant to any one of NVP or EFV. In terms of the mutations sites among these 38 cases, K103N (60.5%, 23/38) was the most frequent TDR-associated mutation, followed by G190A (21.2%, 8/38).

**Table 2:** Analysis of resistance level against antiretroviral drugs.

Drug	Drug resistance level				
	PLR *n* (%)	LR *n* (%)	IR *n* (%)	HR *n* (%)	TDR *n* (%)
PIs					
ATV	2(0.2)	1(0.1)	1(0.1)	0(0.0)	2(0.2)
DRV	0(0.0)	0(0.0)	0(0.0)	0(0.0)	0(0.0)
FPV	9(0.8)	1(0.1)	0(0.0)	0(0.0)	1(0.1)
IDV	2(0.2)	1(0.1)	1(0.1)	0(0.0)	2(0.2)
LPV	2(0.2)	2(0.2)	0(0.0)	0(0.0)	2(0.2)
NFV	25(2.2)	4(0.3)	1(0.1)	1(0.1)	6(0.5)
SQV	2(0.2)	1(0.1)	0(0.0)	1(0.1)	2(0.2)
TPV	9(0.8)	19(1.6)	0(0.0)	0(0.0)	19(1.6)
NRTIs					
ABC	1(0.1)	2(0.2)	2(0.2)	0(0.0)	4(0.3)
AZT	2(0.2)	8(0.7)	1(0.1)	0(0.0)	9(0.8)
D4T	2(0.2)	7(0.6)	2(0.2)	1(0.1)	10(0.9)
DDI	7(0.6)	3(0.3)	0(0.0)	2(0.2)	5(0.4)
FTC	1(0.1)	0(0.0)	1(0.1)	1(0.1)	2(0.2)
3TC	1(0.1)	0(0.0)	1(0.1)	1(0.1)	2(0.2)
TDF	1(0.1)	1(0.1)	0(0.0)	1(0.1)	2(0.2)
NNRTIs					
DOR	18(1.6)	2(0.2)	2(0.2)	1(0.1)	5(0.4)
EFV	104(9.0)	4(0.4)	12(1.0)	30(2.6)	46(4.0)
ETR	125(10.8)	8(0.7)	2(0.2)	0(0.0)	10(0.9)
NVP	111(9.6)	7(0.6)	5(0.4)	38(3.3)	50(4.3)
RPV	111(9.6)	19(1.6)	3(0.3)	2(0.2)	24(2.1)

PLR, potential low resistance; LR, low-level resistance; IR, intermediate-level resistance; HR, high-level resistance; ATV, atazanavir; DRV, darunavir; FPV, fosamprenavir; IDV, indinavir; LPV, lopinavir; NFV, nelfinavir; SQV, saquinavir; TPV, tipranavir; ABC, abacavir; AZT, zidovudine; D4T, stavudine; DDI, didanosine; FTC, emtricitabine; 3TC, lamivudine; TDF, tenofovir; DOR, doravirine; EFV, efavirenz; ETR, etravirine; NVP, nevirapine; RPV, rilpivirine.

### Drug resistance-associated transmission cluster analysis

**Figure 3:**
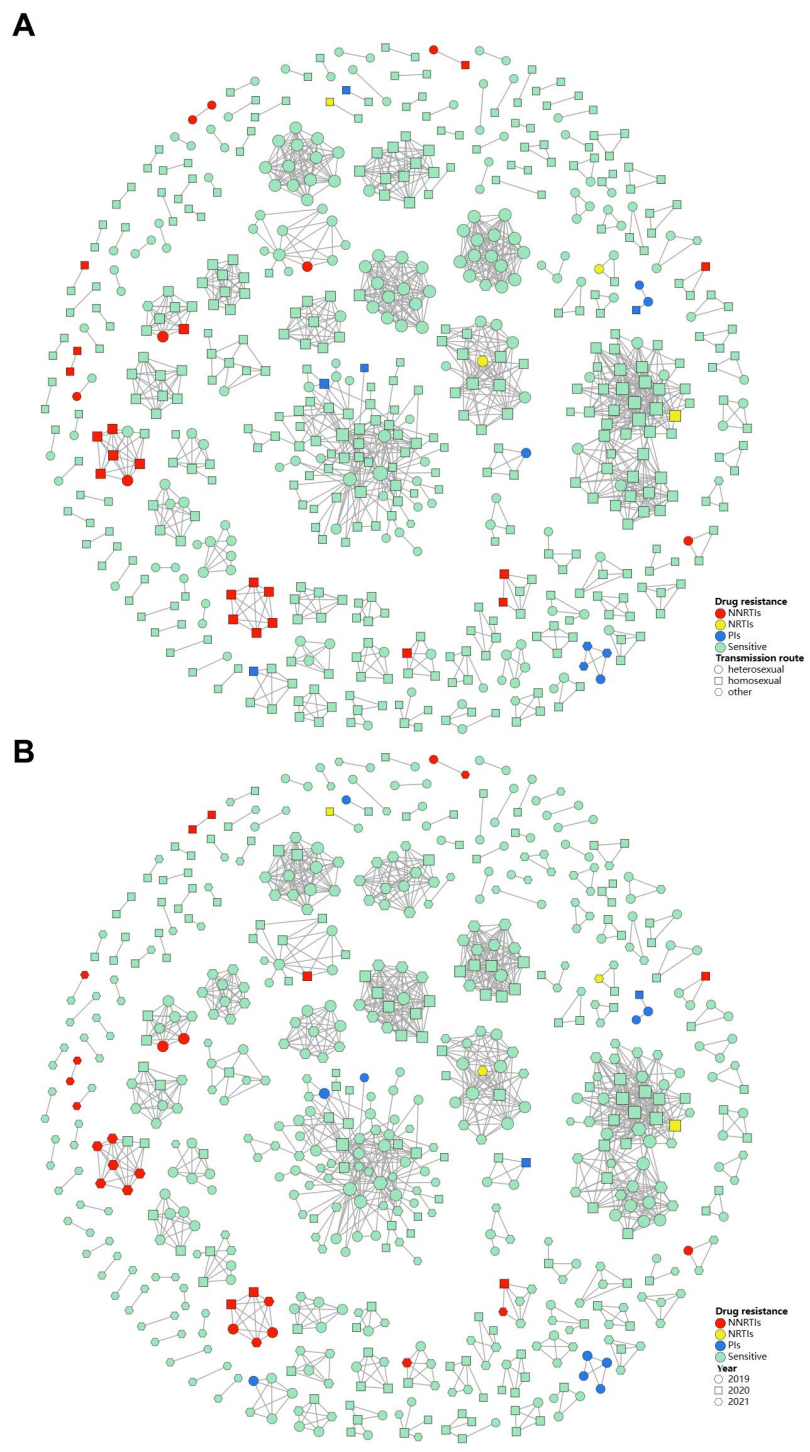
Drug resistance within HIV-1 transmission clusters for different transmission route **(A)** and different year **(B)**. PIs, protease inhibitors; NRTIs, nucleoside reverse transcriptase inhibitors; NNRTIs, non-nucleoside reverse transcriptase inhibitors; sensitive, sensitive to antiretroviral drugs.

We constructed a HIV-1 transmission network using a total of 1,161 sequences (Figure 3). Under the optimal threshold of 1.5% genetic distance, 613 (52.8%) sequences were segregated into 137 clusters in the transmission network, ranging from 2 to 74 sequences. Of the sequences in the network, 40.8% (250/613) were CRF07_BC and 33.9% (208/613) were CRF01_AE. We found that 66.4% (407/613) occurred in homosexual individuals and 32.3% (198/613) in heterosexual individuals. In the largest cluster and the second largest cluster (consisting of 74 individuals and 46 individuals, respectively), MSM was the major route of transmission (71.6 and 89.1%, respectively; Figure 3A).

We identified that 44 TDR cases (48.4%, 44/91) were included in 21 transmission clusters, of which 43.2% (19/44) were primarily concentrated in 4 clusters. Among the 44 TDR cases, 12 cases had resistance to PIs, 4 to NRTIs and 28 to NNRTIs. The majority were male (84.1%), the major subtypes were CRF01_AE (34.1%), CRF07_BC (34.1%) and CRF105_0107 (15.9%), and the major transmission route was homosexual transmission (61.4%). Univariate and multivariate logistic regression analyses were used to explore the factors associated with TDR in the transmission clusters. Female (aOR = 2.692; 95%CI: 1.112–6.521) and ethnic minorities (aOR = 6.752; 95%CI: 1.601–28.475) were associated with higher risk for TDR in clusters (Table 3).

**Table 3:** Factors associated with drug resistance within HIV-1 transmission clusters.

Variables	*N*	Persons in TC (%)	DR in TC(%)	OR (95% CI)	*P*	Adjusted OR (95% CI)	*P*
Sex							
Male	1,090	569(52.2)	37(6.5)	1.000		1.000	
Female	71	44(62.0)	7(15.9)	2.720(1.135–6.518)	0.025	2.692(1.112–6.521)	0.028
Age (yrs)							
16–24	346	182(52.6)	14(7.7)	1.000			
25–49	595	296(49.7)	23(7.8)	1.011(0.506–2.019)	0.975		
≥50	220	135(61.4)	7(5.2)	0.656(0.257–1.673)	0.378		
Marital status							
Single	724	366(50.6)	26(7.1)	1.000			
Married	336	190(56.5)	13(6.8)	0.960(0.482–1.915)	0.909		
Divorced/widowed	101	57(56.4)	5(8.8)	1.257(0.462–3.420)	0.654		
Ethnicity							
Han	1,137	604(53.1)	41(6.8)	1.000		1.000	
Ethnic minorities	24	9(37.5)	3(33.3)	6.866(1.657–28.453)	0.008	6.752(1.601–28.475)	0.009
Education							
Junior or below	257	155(60.3)	14(9.0)	1.000			
Senior	209	105(50.2)	5(4.8)	0.504(0.176–1.443)	0.202		
College or above	695	353(50.8)	25(7.1)	0.768(0.388–1.520)	0.448		
Transmission route							
Homosexual	800	407(50.9)	27(6.6)	1.000			
Heterosexual	350	198(56.6)	16(8.1)	1.237(0.650–2.354)	0.516		
Other	11	8(72.7)	1(12.5)	2.011(0.239–16.942)	0.521		
Diagnosed with STDs except for HIV							
No	812	436(53.7)	30(6.9)	1.000			
Yes	201	103(51.2)	8(7.8)	1.140(0.506–2.565)	0.752		
Unknown	148	74(50.0)	6(8.1)	1.194(0.479–2.977)	0.703		
Screening source							
VCT	610	297(48.7)	21(7.1)	1.000			
Medical institution	325	186(57.2)	12(6.5)	0.906(0.435–1.889)	0.793		
Sexually transmitted disease clinic	130	71(54.6)	4(5.6)	0.785(0.261–2.362)	0.666		
Other	96	59(61.5)	7(11.9)	1.769(0.716–4.375)	0.217		
Baseline CD4 cell count (cells/μL)							
<200	260	117(45.0)	5(4.3)	1.000			
200–499	654	361(55.2)	25(6.9)	1.667(0.623–4.457)	0.309		
≥500	247	135(54.7)	14(10.4)	2.592(0.904–7.428)	0.076		
Baseline viral load before ART (copies/ml)							
<10,000	266	138(51.9)	14(10.1)	1.000			
10,000–99,999	608	308(50.7)	21(6.8)	0.648(0.319–1.316)	0.230		
≥100,000	287	167(58.2)	9(5.4)	0.505(0.211–1.204)	0.123		
Subtype							
CRF01_AE	446	208(46.6)	15(7.2)	1.000			
CRF07_BC	471	250(53.1)	15(6.0)	0.821(0.392–1.722)	0.602		
CRF105_0107	73	63(86.3)	7(11.1)	1.608(0.625–4.139)	0.324		
Subtype B	35	10(28.6)	0(0.0)	-			
CRF55_01B	36	20(55.6)	2(10.0)	1.430(0.303–6.752)	0.652		
Other	100	62(62.0)	5(8.1)	1.129(0.393–3.239)	0.822		
Year							
2019	348	168(48.3)	16(9.5)	1.000			
2020	312	170(54.5)	11(6.5)	0.657(0.296–1.462)	0.303		
2021	501	275(54.9)	17(6.2)	0.626(0.307–1.275)	0.197		

TC, transmission cluster; TDR, transmitted drug resistance; OR, odds ratio; CI, confidence interval; STDs, sexually transmitted diseases; ART, antiretroviral therapy; CRF, circulating recombinant form.

We observed 9 clusters containing interconnected TDR cases (7 clusters with NNRTIs TDR transmission, and 2 clusters with PIs TDR transmission). From 2019 to 2021, we identified 4 clusters that increased in drug-resistant cases during the study period. In addition, one cluster formed in 2019 containing 2 individuals with drug resistance has expanded year by year. This cluster grew to contain 4 individuals in 2020, and 6 in 2021, and all of them within this cluster harbored NNRTIs drug resistance (Figure 3B).

**Figure 4:**
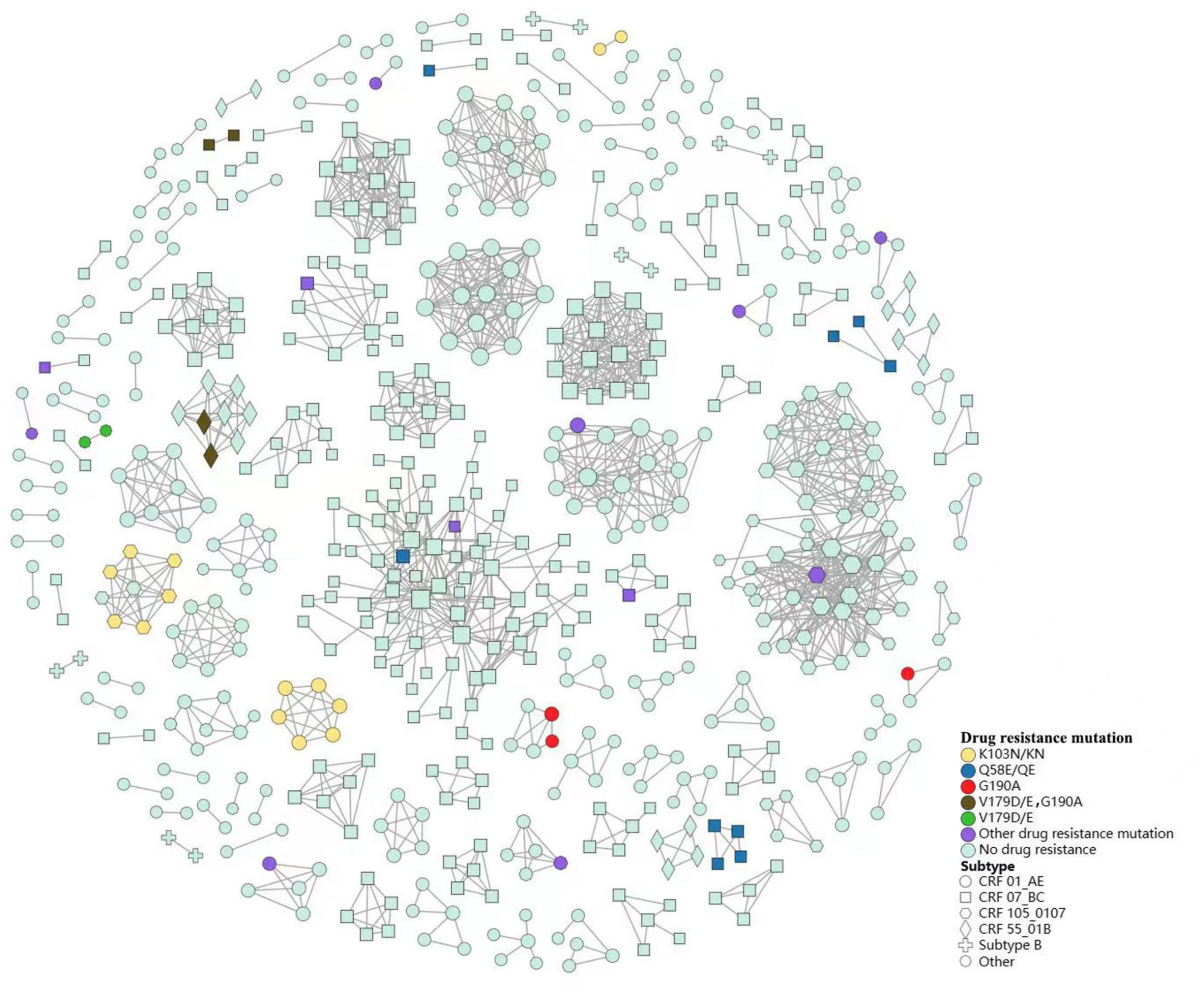
Status of subtype and drug resistance mutations within HIV-1 transmission clusters.

We found that TDR cases were more likely linked to those with the same mutations. Among the 44 TDR cases in transmission clusters, K103N/KN was the most frequent TDR-associated mutation (31.8%, 8 cases in two CRF01_AE clusters and 6 cases in a CRF105_0107 cluster), followed by Q58E/QE (20.5%, 9 cases in four CRF07_BC clusters) and G190A (15.9%, 3 cases in two CRF01_AE clusters, 2 cases in a CRF55_01B cluster and 2 cases in a CRF07_BC cluster). Additionally, 6 cases carried potential low-level resistant mutations (V179D/E; Figure 4).

## Discussion

Our study revealed that the major HIV-1 genotypes detected in Nanjing were CRF07_BC, CRF01_AE and CRF105_01B. Our result was similar to those of the study conducted in Zhejiang province (close neighbor of Jiangsu) and 30 provinces of China (24, 25). In the past 20 years, the distribution of HIV-1 genotypes in Jiangsu Province has undergone changes. During 2000–2001, the predominant subtypes were B′ and C, but they have been replaced by CRF01_AE and CRF07_BC in recent years, meanwhile more URFs produced (26, 27). In this study, the distribution of HIV-1 genotype in Nanjing also changed over time. Since the first detection of a cluster of novel CRF105_0107 HIV-1 strains among MSM in Nanjing in 2017, the prevalence of CRF105_0107 has risen to 6.3% (28), indicating rapid transmission of CRF105_0107 among the population. The proportion of infection with CRF01_AE declined gradually, while that with CRF07_BC increased gradually. The predominant genotype has changed from CRF01_AE in the period of 2015–2019 to CRF07_BC in the study period (29). A series of surveys in various provinces have shown the essential role of CRF07_BC in HIV-1 transmission among MSM (30, 31). CRF07_BC strains were frequently detected in MSM in some parts of China, and its proportion among MSM in Fujian and Guangdong showed an increasing trend in recent years (17, 31, 32). The dominance of CRF07_BC is likely to be maintained if HIV spread rapidly among MSM. Therefore, our study highlighted the importance of CRF07_BC and CRF105_0107 in HIV control in Nanjing. In addition, we observed some unconfirmed unique recombinant strains, suggesting that the changes and complexity of the distribution of HIV-1 genotype has posed a great challenge for HIV prevention and control in Nanjing.

The overall prevalence of TDR in Nanjing was at the moderate level according to the categorization method of WHO (33). The prevalence remained stable during 2019–2021, and higher than the average level of Jiangsu Province during 2009–2011 (3.2%) (27), as well as some regions of China, such as Beijing and Guangdong (17, 34). Besides, it was almost twice the level (4.02%) of a local survey conducted during 2015–2019 (29). The increase in TDR will affect the antiretroviral therapy and the spread of drug resistance. Therefore, we should carry out in-depth investigation and analysis on the causes of TDR increase from the aspects of drug compliance, late diagnosis, mobility of high-risk groups, etc. Meanwhile, we should strengthen the surveillance of TDR and develop strategy to curb the TDR, such as customized ART prescription based on drug resistance prior to ART. Of the 5 main subtypes, the risk of TDR was almost 3 times greater for subtype B than for CRF01_AE, which was consistent with previous results (35, 36). According to a study of 579 cases with ART failure in Jiangsu Province, patients with subtype B infection were associated with higher incidence of drug resistance compared with those infected with CRF07_BC (37), indicating that patients with drug resistance due to ART failure may transmit their own drug resistant strains to the newly infected individuals. The level of TDR in the subtype B was far above the threshold of the high prevalence level of 10%, therefore, non-first-line regimen, such as integrase inhibitors, could be considered among this population.

We found a significantly higher prevalence of mutations related to NNRTI resistance than PIs or NRTIs resistance. The accelerated development and spread of resistance to NNRTIs is not surprising given the strong selection pressures caused by the long-term use of a limited number of NNRTI drugs (38). The distribution of mutation sites was complex. The most common DRMs were V179E and V179D for NNRTIs; M41L and M41ML for NRTIs; Q58E and L33F for PIs. Previous study has shown that in CRF01_ AE, the potential drug resistance mutations V179D/E can synergistically interact with some polymorphic sites, ultimately leading to the transmission of this mutation site in untreated patients (39). As to genotypes, most V179E mutations were detected in CRF55_01B, which was consistent with the research in Guangzhou and Southwest China (13, 40). Therefore, it is speculated that the V179E mutation may be a polymorphism site of CRF55_01B. In China, the combinatorial therapy with TDF/AZT + 3TC + EFV/NVP has been prescribed as the first-line ART regimen because of the limited availability of drugs. Of note, the TDR prevalence of EFV and NVP, the common two kinds of NNRTI drugs, reached 4%. Moreover, the above two drugs mainly exhibited high-level resistance. In this study, the high-level resistance to EFV and NVP primarily resulted from the mutations K103N, which was similar to previous research (41). These findings highlight the importance of identifying HIV-1 resistance patterns at diagnosis or baseline to guide appropriate choice of antiviral therapy. At present, antiretroviral therapy is free in China, but drug resistance testing is expensive and difficult for patients to afford. Therefore, we hope that the government can introduce policies, such as reducing or subsidizing a portion of the testing fee, or making the testing fee covered by medical insurance.

Of the transmission clusters constructed based on obtained HIV-1 sequences, more than three-quarters of the largest two clusters were comprised of sequences from MSM. Nearly half of the individuals infected with drug resistant strains were included in 21 clusters, in which nearly two-thirds of drug resistance was associated with homosexuality. These results illustrated that MSM may contribute essentially to the spread of HIV in Nanjing, and more efforts should be paid to this group. Additionally, the drug-resistant individuals shared a transmission relationship, and the drug-resistant individuals within 4 transmission clusters has increased over time, indicating that the spread of drug resistant strains has occurred in the transmission network, and similar results have been observed in some regions of China (13, 25, 42). In terms of TDR-associated mutations in transmission clusters, K103N/KN and Q58E/QE were the most frequent TDR-associated mutations. They were mainly in 3 clusters and 2 clusters, respectively, in which they linked with those carrying the same mutations. The above two mutations may have been transmitted for a period and spreading onward in the community. Logistic regression analysis revealed that female, ethnic minorities may be related to higher risk for TDR in transmission clusters. The female cases in the clusters were mainly infected by their positive spouse/fixed sexual partner. They may have more chance of TDR if their positive spouse or fixed partner carry transmission drug-resistant mutations themselves or have acquired drug resistance due to failure of ART. The association of these parameters with TDR transmission should be further investigated and taken into considered when implementing subsequent network-based interventions. Therefore, the transmission pattern of TDR should be monitored routinely and in-depth, and later network-based intervention should focused on the drug-resistant individuals or specific clusters, such as clusters of TDR transmission, or clusters of drug resistant individuals that increase over time.

Our study has several limitations. First, there might be selection bias because the newly diagnosed HIV individuals who did not sign the consent form were not enrolled in our study. Second, participants newly diagnosed with HIV-1 infection enrolled in the study were not newly infected. They may have been infected for a considerable period of time, and the non-resistant strains become the dominant strains in the patients (43), making it impossible to detect drug resistance mutations. Third, our analysis of TDR only concentrated on NRTIs, NNRTIs and PIs, while integrase strand transfer inhibitors (INSTIs) were not included, which became an important part of ART. Recent studies have revealed that despite the low prevalence of INSTI resistance in ART-naive patients in some domestic areas, INSTI resistance has been observed in INSTI-treated patients (44, 45). INSTI resistance monitoring should be considered because prescriptions for INSTI-based regimen are anticipated to increase substantially in the near future.

## Conclusion

In summary, this study of 1,161 ART-naïve HIV-1 patients showed diverse and complex distribution of HIV-1 genotypes and mutation site in Nanjing, with CRF01_AE and CRF07_BC as the predominant genotypes. We illustrated that the overall prevalence of TDR was at a moderate level over 3 years. However, nearly half of individuals with TDR were included in transmission clusters, and some drug-resistant individuals have transmitted in clusters. Therefore, HIV drug resistance prevention, monitoring and response efforts should be sustained to reduce the prevalence and transmission of TDR in Nanjing.

## Data availability statement

The authors acknowledge that the data presented in this study must be deposited and made publicly available in an acceptable repository, prior to publication. Frontiers cannot accept a manuscript that does not adhere to our open data policies.

## Ethics statement

The studies involving human participants were reviewed and approved by the Ethics Committee of Nanjing Center for Disease Control and Prevention. Written informed consent to participate in this study was provided by the participants’ legal guardian/next of kin.

## Author contributions

SW, XinL, and HS were contributed to data collection. CD, HZ, XD, and MQ contributed to laboratory testing. XiaL contributed to quality control. YX and HS were responsible for data analysis and manuscript writing. YX, XiaL, and ZZ contributed to the study design and manuscript revision. All authors contributed to the article and approved the submitted version.

## Funding

This research was funded by National Natural Science Foundation of China (Grant no. 82103896), Nanjing Municipal Key Medical Science and Technology Development Project (Grant no. ZKX19050) and Nanjing Key Medical Specialty Project (Infectious Diseases).

## Conflict of interest

The authors declare that the research was conducted in the absence of any commercial or financial relationships that could be construed as a potential conflict of interest.

## Publisher’s note

All claims expressed in this article are solely those of the authors and do not necessarily represent those of their affiliated organizations, or those of the publisher, the editors and the reviewers. Any product that may be evaluated in this article, or claim that may be made by its manufacturer, is not guaranteed or endorsed by the publisher.
