# Supplementary material for: Transmitted drug resistance and transmission clusters among ART-naïve HIV-1-infected individuals from 2019 to 2021 in Nanjing, China
Source: Front Public Health. 2023 Aug 22;11:1179568. doi: 10.3389/fpubh.2023.1179568 (PMC10478099; doi:10.3389/fpubh.2023.1179568)
Supplement: Supplementary file 1 [file Image_1.pdf]

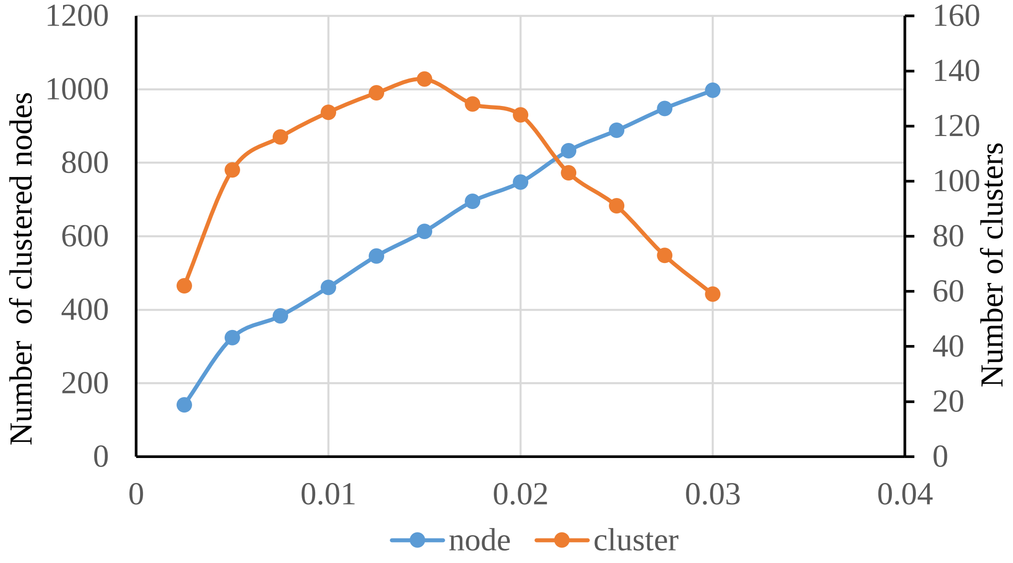

Additional file 1: Figure S1. Evaluation of the effect of the genetic distance threshold on cluster identification.
